# A diary study on the moderating role of leader-member exchange on the relationship between job characteristics, job satisfaction, and emotional exhaustion

**DOI:** 10.3389/fpsyg.2022.812103

**Published:** 2022-10-28

**Authors:** Lennart Poetz, Judith Volmer

**Affiliations:** Work and Organizational Psychology Group, Institute of Psychology, University of Bamberg, Bamberg, Germany

**Keywords:** job characteristics, emotional exhaustion, job satisfaction, leader-member exchange, conservation of resources theory, lagged effects

## Abstract

Job characteristics play an essential role for the well-being of employees. When job characteristics are unfavorable, the experienced exchange relationship with one’s supervisor (i.e., leader-member exchange, LMX) may become relevant to weaken negative consequences. We conducted a diary study over ten consecutive working days with 112 academics. Based on conservation of resources theory, we assumed that daily LMX constitutes a resource for employees that moderates the link between job characteristics (job control and time pressure) and job satisfaction as well as emotional exhaustion. Additionally, we proposed lagged-effects of morning job characteristics and LMX on next-day morning job satisfaction and emotional exhaustion. Findings from hierarchical linear modeling (HLM) demonstrated that on the day-level higher perceived levels of job control in the morning were associated with higher perceived job satisfaction and lower perceived emotional exhaustion in the afternoon. The experience of increased time pressure in the morning was negatively related to perceived day-level afternoon job satisfaction and positively to perceived day-level afternoon emotional exhaustion. Within one day, perceived LMX moderated the relationship between perceived job control and perceived job satisfaction in the afternoon. We only found lagged effects of the interaction between afternoon job control and afternoon LMX on next-day morning job satisfaction. We discuss daily LMX as a resource for employees both within one day and from day-to day, along with future research directions on the buffering role of LMX.

## Introduction

Employee well-being is beneficial both for the individual and the organization because it is related to numerous work-related outcomes such as job performance and productivity (Wright and Cropanzano, 2000; Judge et al., 2001; Alessandri et al., 2017), as well as commitment (Mathieu et al., 2016). Researchers and practitioners have long been interested in how employees’ well-being can be supported and identified several job demands and resources associated with well-being (Bliese et al., 2017). In particular, the characteristics of employees’ jobs have the potential to function either as demands (e.g., time pressure) or as resources (e.g., job control) and are related to well-being (e.g., Crawford et al., 2010).

Job characteristics are not stable but can also vary within one individual from one day to the next (Oerlemans and Bakker, 2018). This variation means that it is essential to study the consequences of job characteristics not only on a general level (e.g., Crawford et al., 2010) but also on a daily, within-person level of analysis. Therefore, compared to general job characteristics, daily job characteristics are stronger related to single activities (Oerlemans and Bakker, 2018). For example, a person who usually experiences high levels of job control and time pressure can still experience lower levels of job control and time pressure within day-specific activities or tasks and vice versa. However, most studies have treated job characteristics as relatively stable over time (Humphrey et al., 2007). Currently, we know very little about the associations of daily job characteristics with fluctuating work-related outcomes. Additionally, it is unclear whether and how these associations transfer onto the next day. This oversight is unfortunate as a static and time-invariant perspective can “inhibit research results, misrepresent reality, and limit the development of a comprehensive body of management knowledge” (McCormick et al., 2020, p. 322), and between-person associations do not necessarily reflect within-person associations (Gabriel et al., 2019). Therefore, we tackle both aspects in the present study. We build our research on the conservation of resources (COR) theory (Hobfoll, 1989) which states that individuals try to keep, protect, and cultivate their resources. Resources may be understood to be “those objects, personal characteristics, conditions, or energies that are valued by the individual or that serve as a means for attainment of these objects, personal characteristics, conditions, or energies” (Hobfoll, 1989, p. 516). Specifically, we examine the relationship between job-related and social resources and work-related outcomes within one day. Additionally, studies on recovery (c.f., Sonnentag et al., 2017) show that events on one day can be associated with behavior, attitudes, and feelings on the next day, which is why we investigate the day-to-day dynamic of work-related experiences and outcomes. By clarifying whether the assumed relationships transfer to the next day (i.e., by assessing potential lagged effects), the present study complements previous research (e.g., Rudolph et al., 2016) and provides insights into the stability of the associations under investigation.

As outcomes, we focus on emotional exhaustion and job satisfaction. Unfavorable job characteristics negatively affect occupational health (Nahrgang et al., 2011) and have been related to burnout (Fernet et al., 2013). Emotional exhaustion is a central indicator of burnout (Maslach et al., 2001) and fluctuates daily (Hülsheger et al., 2013; Volmer and Wolff, 2018; Podsakoff et al., 2019). From an occupational health perspective, there is a need to investigate daily predictors of changes in emotional exhaustion. We propose that day-to-day variability in job characteristics can help explain those daily fluctuations. Additionally, job satisfaction has been considered one of the most important job attitudes (Woznyj et al., 2022). Therefore, and to incorporate also cognitive-affective aspects of work-related outcomes, we investigate whether daily variability in job satisfaction is related to fluctuating job characteristics. Furthermore, job satisfaction is a critical outcome of resource gain and loss (Ten Brummelhuis and Bakker, 2012a) which becomes particularly relevant against the background of varying resources in accordance with varying job characteristics.

We examine daily job control and time pressure as daily job characteristics that can help explain variations in daily emotional exhaustion and job satisfaction. The general level of job control is one of the most important factors for general emotional exhaustion and job satisfaction (Humphrey et al., 2007; Crawford et al., 2010). However, we are unaware of any studies investigating these associations on the day-level. Given the fluctuating nature of job control (Oerlemans and Bakker, 2018), it is crucial to examine the role of daily job control as a job resource for employees’ daily emotional exhaustion and job satisfaction. From a practical perspective, this knowledge can help to design and implement well-grounded job control interventions. Daily time pressure, in turn, is a challenge demand (Cavanaugh et al., 2000) associated with symptoms of strain (e.g., emotional exhaustion; Prem et al., 2018) but also with favorable outcomes (e.g., work engagement or creativity; Ohly and Fritz, 2010; Baethge et al., 2018). However, the double-edged nature of daily time pressure regarding daily job satisfaction is underresearched; therefore, our knowledge of daily time pressure is limited. The lack of research in this area is unfortunate as job satisfaction is a crucial job attitude able to predict behavioral intentions (e.g., turnover intentions) or actual behaviors (e.g., job performance; Woznyj et al., 2022). Between-person findings support the assumption that daily time pressure can benefit employees’ job satisfaction (Podsakoff et al., 2007). This assumption has yet to be tested on the day-level to enlarge the nomological net of daily time pressure and to give practitioners well-informed guidance on the role of time pressure in employees’ everyday work.

Last, we tackle another crucial question for employees’ daily work: what might help them sustain job satisfaction and avoid emotional exhaustion even when confronted with unfavorable working conditions? Depending on the work setting, optimal work design is often not possible, for example, because of external deadlines or in cases where the nature of a task does not allow high levels of autonomy. In a high-quality exchange relationship with a leader, leaders can support their employees with tangible and intangible resources, such as information, professional advice, feedback, acknowledgment, and trust (Martinaityte and Sacramento, 2013). The exchanged resources have been associated with beneficial outcomes for followers and were shown to help them to cope with job demands (Law-Penrose et al., 2016). A follower’s relationship with the leader is one of the most central relationships at work (Thomas et al., 2013), “a critical factor via which many other organizational factors are filtered” (Martinaityte and Sacramento, 2013, p. 977), and high-quality exchanges with the leader can help to create a working environment that addresses the follower’s needs and values (Ellis et al., 2019). Therefore, we propose that the daily experienced exchange relationship with one’s supervisor (i.e., leader-member exchange [LMX]; Graen and Uhl-Bien, 1995) functions as a social resource for employees when facing adverse working conditions. Building on earlier calls (Sonnentag and Pundt, 2016), we test the moderating role of LMX on the link between job characteristics and work-related outcomes. As LMX can vary intra-individually from day to day (Ellis et al., 2019), we focus on its daily buffering role. We conceptualize high LMX as an important daily job resource for followers that can attenuate the negative link between job stressors and outcomes. Thus, we aim to move the literature on leadership and followers’ well-being forward (e.g., Inceoglu et al., 2018) by investigating the leader’s role as a health manager when followers face unfavorable job characteristics.

In sum, we examine the relationship of daily job characteristics (i.e., job control and time pressure) with daily outcomes (i.e., job satisfaction and emotional exhaustion) within a single day and lagging into the next day, as well as the moderating role of daily LMX. We test our hypotheses in a diary study across ten consecutive days (i.e., two standard work weeks) with two measurement points per day in a sample of academics. In the next section, we outline the theoretical reasoning behind our hypotheses.

## Theoretical background and hypotheses

### Daily job characteristics and work-related outcomes

Job control, conceptualized as a job resource, is an important job characteristic describing the range of freedom a person has when carrying out a task. According to Ulich (2011), job control refers to the possibility of deciding on how to solve a task (e.g., time structuring or choice of tools and approaches), the amount of variability one has, and the degree to which a person is allowed to make one’s own decisions about which tasks to work on. Job control can play an important role in emotional exhaustion. Meta-analyses of between-person studies have suggested a negative relationship between job control and burnout, and emotional exhaustion (Crawford et al., 2010; Nahrgang et al., 2011; Park et al., 2014). From a COR theory perspective, job control is a resource that can help protect against impaired well-being. Additionally, dealing with a lack of job control can also be understood as a job demand that is thought to be reflected in increased daily emotional exhaustion (Bakker, 2015). Similarly, several studies have shown a positive association between job control and job satisfaction on a general level (e.g., Humphrey et al., 2007), and there is evidence that job control has a stronger correlation with job satisfaction than any other job characteristic (Loher et al., 1985).

Comparing findings from between-level and within-person levels, we argue that an empirical test is warranted, given the fact that for emotional exhaustion and job satisfaction, respectively, 26% of the within-and between-person correlations of the same constructs were shown to be different from one level to another, with 6% indicating the opposite signs (McCormick et al., 2020). We propose that daily job control constitutes a resource associated with lower daily emotional exhaustion and greater daily job satisfaction. For example, on days employees engage in tasks and activities where they can decide, to a greater degree than on other days, when and how to perform the tasks, they can enjoy these greater degrees of freedom as it gives them the chance to structure and organize themselves in accordance with own preferences, strengths, and competences. Even though deciding how to perform a task might need resources, less effort is required during the performance itself because employees do not need to spend effort on information processing. Less effort should be associated with lower emotional exhaustion at the end of the working day. In sum, we postulate the following hypotheses for the association between daily job control and job outcomes (i.e., emotional exhaustion and job satisfaction).

*H1a*: Daily job control in the morning is negatively related to daily emotional exhaustion in the afternoon.

*H1b*: Daily job control in the morning is positively related to daily job satisfaction in the afternoon.

Time pressure is a condition at work that is, for example, characterized by meeting strict deadlines, working fast, or being under pressure to complete one’s tasks. Within the job demands, job resources framework, time pressure is a job demand for employees. Defined as a situation with too much to do in too little time (Fay and Sonnentag, 2002), time pressure is often understood as a challenge stressor (Cavanaugh et al., 2000). Challenge stressors (vs. hindrance stressors) are those “that people tend to appraise as potentially promoting their personal growth and achievement” (Podsakoff et al., 2007, p. 438). Time pressure was previously conceptualized as a challenge stressor because it can be dealt with by putting more effort into a task. Challenge stressors were argued and found to be a double-edged sword for individuals. Specifically, they were found to be positively related to both strain (e.g., emotional exhaustion) and job satisfaction, even though strain and job satisfaction were negatively related (Podsakoff et al., 2007). As such, challenge stressors lead to greater strain but at the same time to positive affective responses.

We align with these findings and hypothesize that daily time pressure shows differential associations with the strain-related (i.e., emotional exhaustion) and attitudinal facet (i.e., job satisfaction) of employees’ work-related experiences. In line with previous day-level findings (Prem et al., 2018; Pindek et al., 2019; Kunzelmann and Rigotti, 2021), we assume that daily time pressure is associated with increased emotional exhaustion. When employees face a great deal of time pressure, they need to put extra effort into their performance to complete their work by working faster or multitasking. Therefore, even demands that are seen as a challenge for employees require and drain their resources and hence are associated with psychological costs and a loss of resources (Kunzelmann and Rigotti, 2021). From a COR perspective, these may be reflected in feelings of increased emotional exhaustion.

Research on the association between daily time pressure and daily job satisfaction is lacking. Nevertheless, based on previous findings and the idea of challenge stressors (Podsakoff et al., 2007), it is reasonable to assume that even though daily time pressure can cause strain, it can at the same time be associated with positive responses, such as greater daily task performance (Binnewies et al., 2009), daily creative and proactive behavior (Ohly and Fritz, 2010), or daily work engagement (Baethge et al., 2018). We expect similarly positive responses for job satisfaction as a crucial job attitude and suggest that daily time pressure is positively connected to daily job satisfaction. The efforts that go along with time pressure can be related to the perception of meeting demands, facilitating progress toward goals, and feeling personal accomplishment (Kunzelmann and Rigotti, 2021). This process is also in line with COR theory, which proposes that individuals must invest resources to gain resources in the future. Therefore, time pressure as a job demand might trigger the investment of additional resources. In turn, successfully satisfying demands can go along with feelings of achievement due to an increased amount of finished work in one day or the perception that one has a demanding job that requires the use of personal strengths and competencies. Feelings of achievement and self-competence are positive and can be interpreted as important resources for employees (Hobfoll et al., 2018). These states may, in turn, be reflected in increased job satisfaction.

Therefore, based on our theoretical argumentation and previous findings showing the double-edged nature of time pressure, we assume that daily time pressure is at the same time harmful for employees concerning the experience of strain and beneficial for them regarding their job attitude. We hypothesize the following:

*H1c*: Daily time pressure in the morning is positively related to daily emotional exhaustion in the afternoon.

*H1d*: Daily time pressure in the morning is positively related to daily job satisfaction in the afternoon.

### Job characteristics and next-day work-related outcomes

In addition to within-day relationships, we also make predictions about lagged effects from one day to the next. Most studies of the lagged effects of job stressors on well-being have examined them over 1-or 2-year timespans. Indeed, few have investigated these relationships from a short-term (i.e., within one day) or mid-term (i.e., within weeks) perspective. However, others showed that studying short-term and mid-term effects of stressors on work-related outcomes is important, as mid-term reactions to stressors can result from repeated daily stressors (Dudenhoeffer and Dormann, 2013). These reactions might, in turn, elicit long-term stress outcomes.

Research into same-and next-day relationships is more settled regarding recovery experiences or behavior at work, and there is support for the assumption that events on one day are associated with those on the next (e.g., Fritz and Sonnentag, 2009; Ten Brummelhuis and Bakker, 2012b; Nicholson and Griffin, 2015). Other studies have demonstrated that job characteristics (i.e., hindrance stressors, challenge stressors, and job resources) explain a broader variance in evening measures of well-being than do recovery experiences (Bennett et al., 2018), and that evening recovery is linked to next-day behavior and well-being (e.g., Chawla et al., 2020). We argue that job characteristics on one day are not only related to same-day but also next-day outcomes. We build on the resource gain spirals that are proposed in COR theory. These refer to a positive cycle of possessing, investing, and building resources, which means that those with available resources have a greater chance of coping with demands and investing and building new resources. The opposite is the case for those who lack resources. They are more prone to a loss spiral, meaning that resources are threatened or lost, which impedes resource replenishment (Hobfoll, 1989; Ellis et al., 2019). As outlined above, we suggest that employees acquire resources when facing more favorable job characteristics on a particular day. These resource gains are reflected in greater job satisfaction and lower emotional exhaustion on that day. Resourceful states on one day give employees the chance to enter a resource gain spiral, which increases the likelihood of them keeping or even increasing their resources across time (i.e., into the next day). Therefore, we assume that employees who feel more satisfied with their job and less emotionally exhausted on one day will also keep their level of job satisfaction or emotional exhaustion until the next morning. Events and behaviors on one day potentially enhance employees’ psychological functioning and their pool of resources (Steger et al., 2008). Additionally, this resource-rich state can signal to individuals that the working environment allows them to acquire resources and is associated with beneficial outcomes. Therefore, such a positive reflection of one’s work (i.e., experiencing greater job satisfaction and less emotional exhaustion) can trigger a virtuous cycle so that increased job satisfaction and reduced emotional exhaustion is not restricted to one day only.

*H2a*: Daily morning job control on one day is negatively related to daily morning emotional exhaustion on the following day.

*H2b*: Daily morning job control on one day is positively related to daily morning job satisfaction on the following day.

*H2c*: Daily morning time pressure on one day is positively related to daily morning emotional exhaustion on the following day.

*H2d*: Daily morning time pressure on one day is positively related to daily morning job satisfaction on the following day.

### The moderating role of LMX

Research has shown that, compared with other leadership constructs, LMX was best suited to predict well-being (Gregersen et al., 2014). Additionally, LMX is viewed as an important work-related social resource that can reduce employees’ negative experiences (Erdogan and Bauer, 2014; Zhang et al., 2019). Therefore, LMX is an essential variable when studying leadership and work-related outcomes. LMX theory refers to the quality of the relationship between the supervisor and the follower. Its roots lie in role theory (Graen and Scandura, 1987) and social exchange theory (c.f., Dulebohn et al., 2012) and argues that leaders develop unique and differential relationships with every follower. It has been criticized recently (Gottfredson et al., 2020; Scandura and Meuser, 2022) for being ill-defined, as drawing a weak distinction between antecedents and consequences (e.g., trust, liking, or other leadership constructs), and being misaligned in terms of conceptualization and measurement. We acknowledge these criticisms and discuss LMX’s limitations in the Discussion section.

Most research on LMX has investigated its direct or mediating effects. For example, Gottfredson and Aguinis (2017) studied the leadership behavior–follower performance link and revealed that LMX acted as a mediator. The authors concluded that LMX is an underlying mechanism that can explain how certain leadership behaviors lead to particular performances by followers. However, we argue that the focus should not only be on the direct link between LMX and work-related outcomes but also on potential moderating effects. Scholars proposed five ways leadership can influence employee well-being (Wegge et al., 2014). For instance, they suggested that leaders “act as a buffer against high levels of demands at work or as a factor that serves to mobilize existing resources” (Wegge et al., 2014, p. 12). Leaders might play a role in determining how certain (stressful) work situations are interpreted by their followers (e.g., a high workload can either be seen as a threat or a challenge) or help them use existing resources. We consider a high-quality LMX relationship to be an important resource for employees (Lee and Ashforth, 1996; Breevaart et al., 2015; Nielsen et al., 2017; Zhang et al., 2019) that can both support the conservation of resources and the acquisition of new ones. We are therefore responding to recent calls for more research on the moderating role of LMX (Wegge et al., 2014; Sonnentag and Pundt, 2016).

Few studies have investigated LMX as a moderator. It has been found to attenuate the negative relationship between job-related stressors and different outcome measures such as job satisfaction and organizational citizenship behavior (Hackney et al., 2018) or work–family conflict (Zhang et al., 2019). In these cases, LMX was considered a resource that buffers the negative link between work stressors and different outcome variables. However, the two studies mentioned above differ from the present study because they investigated the constructs at a general, between-person level. On the other hand, we focus on daily LMX as a moderating variable. Recent research has demonstrated that leadership in general (Kelemen et al., 2020) and leader-follower interactions—and LMX in particular (Volmer, 2015; Ellis et al., 2019; Richter-Killenberg and Volmer, 2022)—vary substantially within individuals from day to day. Therefore, we examine whether the daily perception of higher- or lower-quality LMX buffers or facilitates the relationship between daily job characteristics and daily job satisfaction and emotional exhaustion.

We acknowledge that leaders are likely to have an impact on job characteristics (Wegge et al., 2014) that would contradict the premise of predictor and moderator independence necessary for moderation.^1^ However, we propose that LMX and job characteristics are less closely related on a daily level; it is more likely that the latter are shaped by external factors, such as tasks that have to be carried out in a particular way or external deadlines that create time pressure. In such situations, a follower’s perception of the general LMX might be less important than the quality of the relationship they perceive on a specific day. According to COR theory, days with more unfavorable job characteristics are likely to be associated with actual or expected resource losses related to decreased well-being (e.g., Baethge et al., 2018). To protect against losses, individuals build on resources from their environment (Hobfoll, 1989) – and the quality of the relationship with their leader in the current day is one of the most important (Erdogan and Bauer, 2014; Zhang et al., 2019). In particular, when confronted with lower job control, employees may perceive decreased job satisfaction and increased emotional exhaustion. They may have to invest more resources than usual to perform a task because they have to change how they work. In such a situation, they may benefit from the perception of having a better LMX if their leader trusts them more or provides them with more information or feedback compared with other days. In terms of the daily level, it does not matter how much trust, information, or feedback the employee generally receives; more important is that they can call on more resources than on other days, which can help protect against potential resource loss. Similarly, when confronted with higher time pressure, the perception that they are being given more support than on other days can help them perceive such pressure as a challenge. This, in turn, can even facilitate resource gains associated with successfully dealing with demands (i.e., increased job satisfaction; Kunzelmann and Rigotti, 2021). At the same time, it can buffer the losses induced by the need to invest more resources to deal with the increased time pressure (i.e., decreased emotional exhaustion).

*H3a*: Daily LMX moderates the relationship between daily job control and daily emotional exhaustion, such that the relationship will be stronger for low (vs. high) LMX.

*H3b*: Daily LMX moderates the relationship between daily job control and daily job satisfaction, such that the relationship will be stronger for low (vs. high) LMX.

*H3c*: Daily LMX moderates the relationship between daily time pressure and daily emotional exhaustion, such that the relationship will be stronger for low (vs. high) LMX.

*H3d*: Daily LMX moderates the relationship between daily time pressure and daily job satisfaction, such that the relationship will be stronger for high (vs. low) LMX.

### The moderating role of LMX on next-day work-related outcomes

Studies reporting the lagged effects of LMX at the daily or weekly level are scarce. One study found a direct negative link between LMX on one day and exhaustion on the next day, concluding that a good relationship with a leader enhances employee well-being and even persists until the next day (Ellis et al., 2019). Another study demonstrated that high-quality LMX attenuated the detrimental effects of feelings of violation at work during one week and undesirable work-related outcomes during the next (Griep et al., 2016). Based on their finding that job demands such as time pressure increase the likelihood that employees do not recover sufficiently, scholars pointed out the important role leaders play in enhancing the process of effective employee recovery (Chawla et al., 2020; e.g., by signaling that working in the evening is not expected and that employees should take time to recover). This recovery, in turn, is positively related to next-day job satisfaction and negatively to emotional exhaustion.

It is also important to assess the role of time in resource dynamics from a COR perspective (Hobfoll et al., 2018). Our study focuses on whether the timing of the availability of LMX as a resource is relevant, that is, how long its moderating role lasts. We also tackle another important issue: how and when resource loss or gain spirals can be broken or enhanced, respectively (Hobfoll et al., 2018). We propose that a high-quality LMX constitutes a resource for employees that can buffer reduced job satisfaction or high emotional exhaustion over more than one day. It can also help employees maintain or increase their resource pool within the same day and have it carry over into the next day. We assume that a high-quality LMX on one day effectively breaks a resource loss spiral that could develop through the same day and into the next. It can therefore help protect employees from resource losses. Similarly, when employees face more time pressure on one day compared with another, the perception of a high-quality LMX on that day can even help increase their job satisfaction on that day, thereby facilitating the development of a resource gain spiral

*H4a*: LMX on one day moderates the relationship between daily job control that day and daily emotional exhaustion on the next, such that the relationship will be stronger for low (vs. high) LMX.

*H4b*: LMX on one day moderates the relationship between daily job control that day and daily job satisfaction on the next, such that the relationship will be stronger for low (vs. high) LMX.

*H4c*: LMX on one day moderates the relationship between daily time pressure that day and daily emotional exhaustion on the next, such that the relationship will be stronger for low (vs. high) LMX.

*H4d*: LMX on one day moderates the relationship between daily time pressure that day and daily job satisfaction on the next, such that the relationship will be stronger for high (vs. low) LMX.

## Materials and methods

### Procedure

The data for the present study was part of a larger research project on the relationship between job characteristics, leadership, and well-being. The study consisted of a single paper-pencil questionnaire assessing trait and demographic variables and a daily online questionnaire assessing state variables on a day-level.

Before the daily surveys, participants completed a general survey, which was used to collect demographic data and trait variables of the same constructs also used in the daily measures. Completing the general survey took around 15 min. Voluntariness and anonymity have been assured. To match the data of the different measurement points, participants created a unique code specified at the beginning of every survey. The participants were asked to send back the completed survey to the researchers using an enclosed and stamped return envelope. The week after completing the general survey, the participants started the daily diary assessments using a smartphone provided by the researchers. Participants rated their daily LMX, job characteristics, affective states, satisfaction, and emotional exhaustion on ten consecutive working days. Data collection took place twice a day, before noon (11 am, t1) and before the end of the working day (3 pm, t2). An automatic alarm signal was set up on the smartphone to remind participants to complete the questionnaires. Participants received 25€ in exchange for participating in the study.

### Participants

In total, 112 academics (64% females) participated in the study. The mean age of the participants was 30.37 years (*SD* = 5.29). The participants were working at universities (84.8%) and other research institutions (15.2%) and came from different professions, for example, social and economic sciences (34.8%), linguistic and cultural studies (13.4%), natural sciences (10.7%), engineering (2.7%), or law (1.8%). On average, participants worked in research for 3.63 years (*SD* = 3.45). The majority (73.2%) named a master’s or diploma their highest degree, 13.4% a Ph.D., and 1.8% a habilitation. Only a minority (15.2%) of the participants held a supervisory position. On average, participants were in contact with their supervisor once to twice a day (*M* = 1.69, *SD* = 1.25). The participants answered 940 t1 surveys (response rate of 84%) and 903 t2 surveys (response rate of 81%).

### Measures

All measures were assessed in German.

#### General survey

##### Demographic data

As demographic data age, gender, the highest level of education, occupation, weekly working hours, number of years in research, and several questions on the work at the research institution was assessed. In addition, participants were asked about leadership responsibilities, the duration of cooperation with their supervisor, and the number and duration of contact points with their supervisor per day.

*Job control*. Job control was assessed on a five-point scale with four items of the Instrument for stress-related job analysis (ISTA; Semmer et al., 1999). Sample items include “Normally I can decide for myself how to do my job.” or “Normally my work offers me many opportunities to make my own decisions.” Omega was .79.

##### Time pressure

A four-item scale of the same questionnaire as for job control (Semmer et al., 1999) was used to measure time pressure. Example items are “In general, I am under time pressure.” or “Often I have to leave work late because of too much work.” Omega was .82.

##### Leader-member exchange (LMX)

To measure LMX, we used the German version (Schyns, 2002) of the LMX-7 scale (Graen and Uhl-Bien, 1995). Ratings were given on a five-point scale. The names of the scale points were adapted to the respective item. Examples for the items are “How well does your supervisor understand your professional problems and needs?” or “I have enough confidence in my supervisor to defend his/her decisions.” Omega was .90.

##### Job satisfaction

General satisfaction with the job was assessed with the question “How satisfied are you with your work in general?.” Ratings were given on a seven-point scale ranging from *“extremely dissatisfied”* to *“extremely satisfied.”* Following Kaplan et al. (2009), the named scale points were matched with a face scale (Kunin, 1955) as this proved to be the most balanced measure assessing cognitive as well as affective elements of job satisfaction.

##### Emotional exhaustion

To measure emotional exhaustion, participants were asked to rate the eight items of the Oldenburg Burnout Inventory (OLBI; Demerouti et al., 2003), which has the advantage of using positively as well as negatively phrased items. In the OLBI, affective, physical, and cognitive aspects of exhaustion are covered. Sample items include “The strain of my work is quite bearable.” or “I increasingly feel emotionally exhausted in my work.” Ratings were made on a four-point scale. Omega was .86.

#### Daily diary surveys

##### Job control

Job control was assessed with the same items as in the general survey, with the item formulation adapted to the day level (Ohly and Fritz, 2010) and to the time of the assessment. In particular, “normally” was replaced by “today morning/today afternoon.” Omega ranged from .72 to .90, indicating satisfactory to high internal consistency.

##### Time pressure

Three items of the same measure as in the general survey were used to assess time pressure. The item “Today I had to leave work late because of too much work.” which was used in the general survey, was not used in the daily surveys, as this was usually not answerable at this time of the day. Again, the wording was adapted in the same manner as for job control (Ohly and Fritz, 2010). Internal consistency was good, with an omega ranging from .80 to .87.

##### Leader-member exchange (LMX)

LMX was measured with the same scale as in the general survey. Item wording was adapted to fit the daily assessment. Examples are “Today, my supervisor helped me.” or “Today, I had confidence in my supervisor’s decisions.” Items were specified for the time of assessment: the term “in the morning” was added for t1 and “in the afternoon” for t2. Omega ranged from .89 to .94, indicating high internal consistency.

##### Job satisfaction

Current satisfaction with work was assessed with the question “How satisfied are you currently with your work?” The same rating scale as in the general survey was used.

##### Emotional exhaustion

Seven items of the same measure as in the general survey were used to measure emotional exhaustion. The item “After work, I am still fit for my leisure activities.” which was used in the general survey, was not used in the daily surveys as this was usually not answerable at the time of the assessment. The phrasing of the items was adapted to the day level (e.g., Volmer and Fritsche, 2016) and to the time of the assessment by adding “today morning/ today afternoon.” Omega ranged from .79 to .87, indicating good internal consistency.

### Analytic strategy

There are two levels of analysis in a diary study: the person-level (Level 2) and the day-level (Level 1). Person-level data refer to those measures, which are thought not to vary over time, such as demographic data and trait variables. In the current study, all the variables assessed with the general survey can be considered person-level variables. In contrast, all the variables assessed with the daily diary survey have been measured on the day-level. They are presumed to differ intraindividually across the measurement points. To consider the multilevel structure of the data, we applied hierarchical linear modeling (HLM; Raudenbush and Bryk, 2002). HLM takes into account that observations in the data set are not independent of each other. This is important as the day-level measurements are nested within each person (Snijders and Bosker, 2012).

We analyzed the data using the programs IBM SPSS Statistics 25 and HLM 7.03 (Raudenbush et al., 2011). We centered person-level and control variables at the grand mean and day-level predictors at the respective person-mean, the mean across days for each person, as the focus was on within-person effects. To account for unobserved heterogeneity and in line with previous research and recommendations, we specified our Level 1 coefficients as random (Raudenbush and Bryk, 2002; Raudenbush et al., 2011; Bell et al., 2019). As HLM does not allow missing data on Level 2, we replaced missing values on age, gender, trait job satisfaction, and trait emotional exhaustion for one participant in the general survey with the respective scale mean.

Hypotheses testing was done using full maximum likelihood estimation. More specifically, we compared four different models: We started with a null model containing only the intercept. Next, we entered control variables (i.e., age, gender, trait job satisfaction respectively, trait emotional exhaustion, and dyad tenure) in Model 1 as previous research reported links between age (Schulte, 2006) and gender (Clark, 1997) with job satisfaction and of dyad tenure with LMX (Bernerth et al., 2018). Our core model for testing hypotheses 1a-d is Model 2, for which we entered the predictor variables t1 time pressure, respectively, t1 job control. For testing hypotheses 3a-d, Model 4 was of interest, containing both the direct effect of t1 LMX (Model 3) as well as the t1 LMX by t1 time pressure, respectively, the t1 LMX by t1 job control interaction. We tested hypotheses 2a-d similarly to testing hypothesis 1 using Model 2, except that the next day t1 variables were used as dependent variables. The test of hypotheses 4a-d was done likewise to testing hypothesis 3 using Model 4, except that the next day t1 variables were used as dependent variables.

Tables 1–8 show results from HLM analyses, including standardized parameter estimates, variance components for all models, model fit information (i.e., −2*log, deviance values), and differences between the deviance values of the different models (i.e., difference of −2*log). The difference is submitted to a *χ*^2^-test. A significant decline in the deviance, by adding a predictor variable, indicates an improved model fit compared to the previous model.

**Table 1:** Multilevel regression analysis predicting same day afternoon job satisfaction.

	Null model			Model 1			Model 2			Model 3			Model 4		
	Est	SE	*t*	Est	SE	*t*	Est	SE	*t*	Est	SE	*t*	Est	SE	*t*
Intercept	5.00***	0.09	54.14	5.00***	0.06	82.29	5.01***	0.06	82.53	5.03***	0.07	76.74	5.03***	0.07	76.72
Age^a^				0.01	0.01	0.84	0.01	0.01	0.76	0.01	0.02	0.72	0.01	0.02	0.60
Gender^a^				−0.05	0.15	−0.32	−0.05	0.15	−0.32	−0.00	0.17	−0.02	−0.05	0.17	−0.28
Dyad Tenure^a^				0.01	0.03	0.25	0.01	0.03	0.32	0.01	0.04	0.26	0.02	0.04	0.51
Trait Job satisfaction^a^				0.65***	0.07	9.87	0.65***	0.06	10.21	0.66***	0.06	10.26	0.67***	0.07	10.52
Job control^b^							0.21***	0.05	4.25	0.21***	0.06	3.48	0.20**	0.06	3.29
LMX^b^										0.05	0.04	1.24	0.03	0.04	0.77
Job control*LMX^b^													−0.09*	0.05	−2.03
−2*log (lh)		2199.26			2107.29			1922.03			1323.88			1317.28	
Difference of−2*log (lh)					91.97***			185.26***			598.15***			6.60	
df^c^					4			3			4			5	
Level 1 intercept variance (SE)		0.48 (0.02)			0.48 (0.02)			0.42 (0.02)			0.43 (0.03)			0.42 (0.03)	
Level 2 intercept variance (SE)		0.88 (0.13)			0.35 (0.06)			0.35 (0.05)			0.32 (0.06)			0.33 (0.06)	

a Predictors at the person-level.

b Predictors at the day level.

c df refers to the number of parameters added to the model.

* *p* < 0.05;

** *p* < 0.01;

*** *p* < 0.001.

**Table 2:** Multilevel regression analysis predicting same day afternoon job satisfaction.

	Null model			Model 1			Model 2			Model 3			Model 4		
	Est	SE	*t*	Est	SE	*t*	Est	SE	*t*	Est	SE	*t*	Est	SE	*t*
Intercept	5.00***	0.09	54.14	5.00***	0.06	82.29	5.01***	0.06	82.57	5.03***	0.07	76.67	5.03***	0.07	76.76
Age^a^				0.01	0.01	0.84	0.01	0.01	0.85	0.01	0.01	0.82	0.01	0.01	0.76
Gender^a^				−0.05	0.15	−0.32	−0.04	0.15	−0.25	0.04	0.17	0.24	0.03	0.17	0.19
Dyad Tenure^a^				0.01	0.03	0.25	0.00	0.03	0.13	−0.00	0.03	−0.07	0.00	0.04	0.02
Trait Job satisfaction^a^				0.65***	0.07	9.87	0.65***	0.07	9.97	0.65***	0.07	9.74	0.66***	0.07	9.83
Time pressure^b^							−0.12**	0.04	−2.88	−0.11*	0.05	−2.18	−0.10*	0.05	−2.02
LMX^b^										0.05	0.04	1.07	0.06	0.04	1.46
Time pressure*LMX^b^													0.01	0.04	0.18
−2*log (lh)		2199.26			2107.29			1971.91			1360.03			1353.41	
Difference of−2*log (lh)					91.97***			135.38***			611.87***			6.63	
df^c^					4			3			4			5	
Level 1 intercept variance (SE)		0.48 (0.02)			0.48 (0.02)			0.44 (0.02)			0.47 (0.03)			0.45 (0.03)	
Level 2 intercept variance (SE)		0.88 (0.13)			0.35 (0.06)			0.34 (0.05)			0.32 (0.06)			0.32 (0.06)	

a Predictors at the person-level.

b Predictors at the day level.

c df refers to the number of parameters added to the model.

* *p* < 0.05;

** *p* < 0.01;

*** *p* < 0.001.

**Table 3:** Multilevel regression analysis predicting same day afternoon emotional exhaustion.

	Null model			Model 1			Model 2			Model 3			Model 4		
	Est	SE	*t*	Est	SE	*t*	Est	SE	*t*	Est	SE	*t*	Est	SE	*t*
Intercept	2.04***	0.04	51.63	2.04***	0.03	64.45	2.04***	0.03	63.34	2.04***	0.04	58.15	2.04***	0.04	58.22
Age^a^				−0.01	0.01	−1.39	−0.01	0.01	−1.63	−0.01	0.01	−1.26	−0.01	0.01	−1.47
Gender^a^				−0.04	0.08	−0.45	−0.04	0.08	−0.54	−0.02	0.09	−0.21	−0.00	0.08	−0.02
Dyad Tenure^a^				0.02	0.02	1.43	0.02	0.02	1.59	0.01	0.02	0.81	0.01	0.02	0.82
Trait Emotional exhaustion^a^				0.42***	0.06	7.53	0.41***	0.06	7.10	0.42***	0.06	7.00	0.43***	0.06	7.39
Job control^b^							−0.13***	0.03	−4.76	−0.12***	0.03	−3.46	−0.13***	0.03	−3.63
LMX^b^										0.04	0.03	1.40	0.05	0.03	1.79
Job control*LMX^b^													−0.01	0.03	−0.29
−2*log (lh)		1391.47			1341.30			1235.50			834.20			830.63	
Difference of−2*log (lh)					50.17***			105.79***			401.31***			3.56	
df^c^					4			3			4			5	
Level 1 intercept variance (SE)		0.22 (0.01)			0.22 (0.01)			0.21 (0.01)			0.21 (0.01)			0.20 (0.01)	
Level 2 intercept variance (SE)		0.15 (0.02)			0.08 (0.01)			0.09 (0.02)			0.08 (0.02)			0.08 (0.02)	

a Predictors at the person-level.

b Predictors at the day level.

c df refers to the number of parameters added to the model.

*** *p* < 0.001.

**Table 4:** Multilevel regression analysis predicting same day afternoon emotional exhaustion.

	Null model			Model 1			Model 2			Model 3			Model 4		
	Est	SE	*t*	Est	SE	*t*	Est	SE	*t*	Est	SE	*t*	Est	SE	*t*
Intercept	2.04***	0.04	51.63	2.04***	0.03	64.45	2.04***	0.03	63.52	2.04***	0.03	58.11	2.04***	0.04	58.07
Age^a^				−0.01	0.01	−1.39	−0.01	0.01	−1.48	−0.01	0.01	−1.30	−0.01	0.01	−1.25
Gender^a^				−0.04	0.08	−0.45	−0.04	0.08	−0.45	0.01	0.09	0.15	0.01	0.09	0.11
Dyad Tenure^a^				0.02	0.02	1.43	0.02	0.02	1.54	0.01	0.02	0.58	0.01	0.02	0.51
Trait Emotional exhaustion^a^				0.42***	0.06	7.53	0.41***	0.06	7.14	0.43***	0.06	6.95	0.43***	0.06	7.04
Time pressure^b^							0.12***	0.02	4.81	0.13***	0.03	4.64	0.13***	0.03	4.98
LMX^b^										0.03	0.02	1.04	0.02	0.03	0.66
Time pressure*LMX^b^													0.04	0.02	1.74
−2*log (lh)		1391.47			1341.30			1244.67			827.69			824.55	
Difference of-2*log (lh)					50.17***			96.63***			416.98***			3.14	
df^c^					4			3			4			5	
Level 1 intercept variance (SE)		0.22 (0.01)			0.22 (0.01)			0.20 (0.01)			0.20 (0.01)			0.20 (0.01)	
Level 2 intercept variance (SE)		0.15 (0.02)			0.08 (0.01)			0.09 (0.02)			0.08 (0.02)			0.08 (0.01)	

a Predictors at the person-level.

b Predictors at the day level.

c df refers to the number of parameters added to the model.

*** *p* < 0.001.

**Table 5:** Multilevel regression analysis predicting next day morning job satisfaction.

	Null model			Model 1			Model 2			Model 3			Model 4		
	Est	SE	*t*	Est	SE	*t*	Est	SE	*t*	Est	SE	*t*	Est	SE	*t*
Intercept	5.04***	0.08	60.23	5.05***	0.06	88.93	5.05***	0.06	80.33	5.04***	0.07	70.20	5.04***	0.07	70.21
Age^a^				0.01	0.01	0.99	0.01	0.01	0.92	0.02	0.02	1.09	0.02	0.02	1.12
Gender^a^				−0.01	0.14	−0.09	−0.08	0.15	−0.52	−0.15	0.19	−0.8	−0.15	0.19	−0.76
Dyad Tenure^a^				0.00	0.03	0.16	0.02	0.03	0.52	0.03	0.04	0.79	0.23	0.04	0.81
Trait Job satisfaction^a^				0.58***	0.06	9.05	0.56***	0.07	7.94	0.56***	0.08	7.40	0.56***	0.08	7.35
Job control^b^							0.02	0.05	0.44	0.03	0.06	0.47	0.03	0.06	0.53
LMX^b^										0.10*	0.04	2.62	0.08*	0.04	2.18
Job control*LMX^b^													−0.05	0.05	−0.98
−2*log (lh)		2484.30			2397.64			1940.22			1311.59			1308.96	
Difference of−2*log (lh)					86.67***			457.42***			628.63***			2.63	
df^c^					4			3			4			5	
Level 1 intercept variance (SE)		0.62 (0.03)			0.62 (0.03)			0.60 (0.03)			0.56 (0.04)			0.55 (0.04)	
Level 2 intercept variance (SE)		0.70 (0.10)			0.28 (0.05)			0.33 (0.06)			0.35 (0.07)			0.35 (0.07)	

a Predictors at the person-level.

b Predictors at the day level.

c df refers to the number of parameters added to the model.

* *p* < 0.05;

*** *p* < 0.001.

**Table 6:** Multilevel regression analysis predicting next day morning job satisfaction.

	Null model			Model 1			Model 2			Model 3			Model 4		
	Est	SE	*t*	Est	SE	*t*	Est	SE	*t*	Est	SE	*t*	Est	SE	*t*
Intercept	5.04***	0.08	60.23	5.05***	0.06	88.93	5.05***	0.06	80.44	5.04***	0.07	70.14	5.04***	0.07	70.04
Age^a^				0.01	0.01	0.99	0.01	0.01	0.87	0.02	0.02	1.06	0.02	0.02	1.27
Gender^a^				−0.01	0.14	−0.09	−0.07	0.15	−0.49	−0.15	0.19	−0.79	−0.19	0.19	−1.04
Dyad Tenure^a^				0.00	0.03	0.16	0.02	0.03	0.59	0.03	0.04	0.74	0.02	0.04	0.65
Trait Job satisfaction^a^				0.58***	0.06	9.05	0.58***	0.06	9.32	0.56***	0.07	7.89	0.57***	0.07	8.22
Time pressure^b^							−0.02	0.04	−0.41	−0.05	0.05	−0.95	−0.06	0.05	−1.23
LMX^b^										0.11**	0.04	2.92	0.12**	0.04	3.35
Time pressure*LMX^b^													−0.02	0.04	−0.44
−2*log (lh)		2484.30			2397.64			1968.18			1320.79			1318.17	
Difference of−2*log (lh)					86.67***			429.46***			647.38***			2.62	
df^c^					4			3			4			5	
Level 1 intercept variance (SE)		0.62 (0.03)			0.62 (0.03)			0.60 (0.03)			0.56 (0.04)			0.56 (0.04)	
Level 2 intercept variance (SE)		0.70 (0.10)			0.28 (0.05)			0.33 (0.06)			0.35 (0.07)			0.35 (0.07)	

a Predictors at the person-level.

b Predictors at the day level.

c df refers to the number of parameters added to the model.

* *p* < 0.05;

** *p* < 0.01;

*** *p* < 0.001.

**Table 7:** Multilevel regression analysis predicting next day morning emotional exhaustion.

	Null model			Model 1			Model 2			Model 3			Model 4		
	Est	SE	*t*	Est	SE	*t*	Est	SE	*t*	Est	SE	*t*	Est	SE	*t*
Intercept	1.93***	0.04	52.05	1.93***	0.03	65.84	1.93***	0.03	58.61	1.94***	0.04	48.30	1.924***	0.04	48.33
Age^a^				−0.01	0.01	−1.75	−0.01	0.01	−1.50	−0.01	0.01	−1.52	−0.01	0.01	−1.53
Gender^a^				−0.03	0.07	−0.39	−0.02	0.08	−0.22	0.03	0.10	0.32	0.03	0.10	0.32
Dyad Tenure^a^				0.02	0.01	1.10	0.01	0.02	0.78	0.01	0.02	0.49	0.01	0.02	0.51
Trait Emotional exhaustion^a^				0.40***	0.05	7.82	0.37***	0.06	6.39	0.42***	0.07	5.91	0.42***	0.07	5.93
Job control^b^							0.01	0.03	0.38	−0.00	0.04	−0.08	−0.00	0.04	−0.10
LMX^b^										−0.02	0.03	−0.77	−0.02	0.03	−0.69
Job control*LMX^b^													−0.01	0.04	−0.40
−2*log (lh)		1608.70			1556.37			1250.71			871.03			868.62	
Difference of−2*log (lh)					52.33***			305.66***			379.68***			2.41	
df^c^					4			3			4			5	
Level 1 intercept variance (SE)		0.27 (0.01)			0.27 (0.01)			0.26 (0.01)			0.25 (0.02)			0.25 (0.02)	
Level 2 intercept variance (SE)		0.12 (0.02)			0.06 (0.01)			0.06 (0.01)			0.09 (0.02)			0.10 (0.02)	

a Predictors at the person-level.

b Predictors at the day level.

c df refers to the number of parameters added to the model.

*** *p* < 0.001.

**Table 8:** Multilevel regression analysis predicting next day morning emotional exhaustion.

	Null model			Model 1			Model 2			Model 3			Model 4		
	Est	SE	*t*	Est	SE	*t*	Est	SE	*t*	Est	SE	*t*	Est	SE	*t*
Intercept	1.93***	0.04	52.05	1.93***	0.03	65.84	1.93***	0.03	59.23	1.93***	0.04	48.22	1.94***	0.04	48.22
Age^a^				−0.01	0.01	−1.75	−0.01	0.01	−1.44	−0.01	0.01	−1.47	−0.01	0.01	−1.35
Gender^a^				−0.03	0.07	−0.39	−0.01	0.08	−0.13	0.04	0.01	0.40	0.04	0.10	0.44
Dyad Tenure^a^				0.02	0.01	1.10	0.01	0.02	0.80	0.01	0.02	0.48	0.01	0.02	0.47
Trait Emotional exhaustion^a^				0.40***	0.05	7.82	0.38***	0.06	6.74	0.42***	0.07	5.91	0.42***	0.07	6.00
Time pressure^b^							0.04	0.03	1.29	0.07	0.04	1.90	0.07*	0.03	2.19
LMX^b^										−0.02	0.03	−0.86	−0.02	0.03	−0.86
Time pressure*LMX^b^													0.00	0.03	0.12
−2*log (lh)		1608.70			1556.37			1262.13			875.00			871.92	
Difference of−2*log (lh)					52.33***			294.24***			387.13***			3.08	
df^c^					4			3			4			5	
Level 1 intercept variance (SE)		0.27 (0.01)			0.27 (0.01)			0.25 (0.01)			0.25 (0.02)			0.25 (0.02)	
Level 2 intercept variance (SE)		0.12 (0.02)			0.06 (0.01)			0.08 (0.02)			0.10 (0.02)			0.10 (0.02)	

a Predictors at the person-level.

b Predictors at the day level.

c df refers to the number of parameters added to the model.

* *p* < 0.05;

*** *p* < 0.001.

## Results

### Descriptive results

Means, standard deviations, and zero-order correlations of the Level 2 study variables are shown in Table 9. Means, standard deviations, and correlations of the group-mean centered Level 1 study variables are shown in Table 10.

**Table 9:** Means (M), standard deviations (SD) and correlations of Level 2 study variables.

	M	SD	1	2	3	4	5	6	7	8
1. Gender^a^	1.35	0.48	1							
2. Age	30.37	5.29	0.26^**^	1						
3. Dyad tenure	2.86	2.57	0.24^*^	0.52^**^	1					
4. Job control	3.99	0.71	−0.06	−0.12	0.03	1				
5. Time pressure	3.07	0.87	−0.06	0.20^*^	0.05	−0.20^*^	1			
6. LMX	3.58	0.79	−0.11	−0.15	−0.11	0.53^**^	−0.04	1		
7. Job satisfaction	5.23	1.14	−0.05	−0.15	−0.09	0.54^**^	−0.25^**^	0.57^**^	1	
8. Emotional exhaustion	2.19	0.60	0.06	0.09	0.10	−0.44^**^	0.50^**^	−0.38^**^	−0.73^**^	1

a female = 1, male = 2.

* *p* < 0.05;

** *p* < 0.01.

*N* = 112.

**Table 10:** Correlations of group-mean centered Level 1 study variables.

	1	2	3	4	5	6	7	8	9	10
1. JC (m)	1									
2. JC (a)	0.61^**^	1								
3. TP (m)	−0.31^**^	−0.24^**^	1							
4. TP (a)	−0.24^**^	−0.31^**^	0.61^**^	1						
5. LMX (m)	0.01	−0.02	0.07	0.04	1					
6. LMX (a)	0.03	−0.04	0.00	0.11^**^	0.23^**^	1				
7. JS (m)	0.23^**^	0.13^**^	−0.11^**^	−0.11^**^	0.23^**^	0.07	1			
8. JS (a)	0.22^**^	0.25^**^	−0.14^**^	−0.14^**^	0.06	0.22^**^	0.45^**^	1		
9. EXH (m)	−0.25^**^	−0.17^**^	0.27^**^	0.17^**^	−0.04	−0.05	−0.53^**^	−0.38^**^	1	
10. EXH (a)	−0.20^**^	−0.24^**^	0.21^**^	0.29^**^	0.06	−0.03	−0.36^**^	−0.49^**^	0.62^**^	1

n = 584–941. JC, Job control; TP, Time pressure; LMX, Leader-member exchange; JS, Job satisfaction; EXH, Emotional exhaustion. m, assessment in the morning. a, assessment in the afternoon.

* *p* < 0.05;

** *p* < 0.01.

On a descriptive basis, participants reported relatively low daily levels of time pressure and emotional exhaustion but relatively high daily levels of job control and job satisfaction. The reported daily LMX quality was slightly lower than the scale mean. Interestingly, the reported daily LMX quality was around one scale point lower than the reported general LMX quality. At first glance, correlational data mostly supported the assumed direct associations between job characteristics and work-related outcomes within one day. An exception was the time pressure – job satisfaction link, which showed to have a negative instead of a positive direction.

### Preliminary analysis

To use HLM as a method, there needs to be sufficient variance in the Level 1 outcome variables, that is, within-person variance. To examine if this precondition is met, we calculated intra-class correlations (ICC). For job satisfaction, the ICC was 0.65, meaning that 35% of the variance was on the within-person level. The ICC for emotional exhaustion was 0.41, indicating that 59% of the variance was on the within-person level. The ICC for LMX was 0.54, indicating that 46% of the variance was on the within-person level. These results are fairly comparable to the ones that Podsakoff et al. (2019) reported. As the ICC indicates a nontrivial amount of variance on the day-level, the use of HLM for hypotheses testing is justified.

### Hypotheses testing

#### Same day job satisfaction as dependent variable

**Figure 1:**
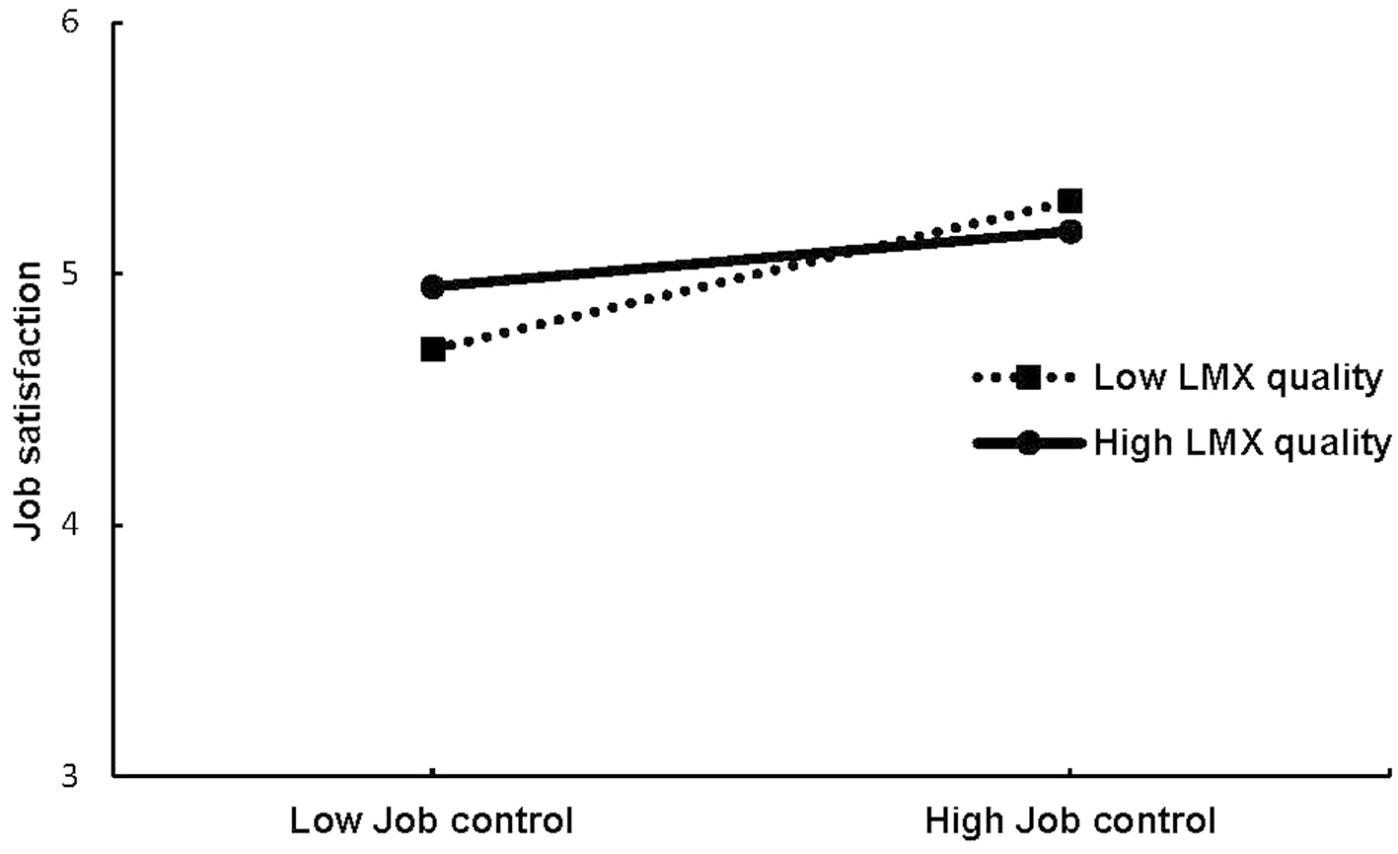
Interaction between daily job control and daily LMX quality on job satisfaction.

Adding control variables to the null model significantly improved the model fit (Δ −2*log = 91.97, *p* < 0.001). Results indicated that only trait job satisfaction was a significant predictor of daily job satisfaction [*γ* = 0.65, *SE* = 0.07, *t*(106) = 9.87, *p* < 0.001]. For the next model (Model 2) we entered the predictor variables job control, respectively, time pressure. The models including job control, respectively, time pressure fit the data better than the previous one (for job control: Δ −2*log = 185.26, *p* < 0.001; for time pressure: Δ −2*log = 135.38, *p* < 0.001). Job control significantly and positively predicted job satisfaction [*γ* = 0.21, *SE* = 0.05, *t*(110) = 4.25, *p* < 0.001], therefore confirming hypothesis 1b as more job control in the morning went along with more job satisfaction in the afternoon. Job satisfaction was also significantly predicted by time pressure [*γ* = −0.12, *SE* = 0.04, *t*(110) = −2.88, *p* < 0.01]. Counter to our hypothesis, both variables were negatively related, therefore hypothesis 1d was not supported. To test hypotheses 3b and 3d, Model 2 was first extended by LMX (Model 3) and then by the LMX by job control, respectively, the LMX by time pressure interaction (Model 4). Compared to Model 3, Model 4 did not show a better model fit (Δ −2*log = 6.6, *p* = 0.25). However, the LMX by job control interaction showed to be a significant predictor of job satisfaction [*γ* = −0.09, *SE* = 0.05, *t*(103) = −2.03, *p* < 0.05]. The nature of the interaction effect is displayed in Figure 1. As can be seen, the relationship between job control and job satisfaction is stronger for low LMX quality. Thus, hypothesis 3b was supported by the data. However, there was no support for hypothesis 3d as including the LMX by time pressure interaction (Model 4) did not result in a better model fit (Δ −2*log = 6.63, *p* = 0.25). Furthermore, the interaction term failed to reach significance [*γ* = 0.01, *SE* = 0.04, *t*(103) = 0.18, *p* = 0.86].

#### Same day emotional exhaustion as dependent variable

When predicting emotional exhaustion, adding control variables to the null model improved the model fit (Model 1; Δ−2*log = 50.17, *p* < 0.001). However, only trait emotional exhaustion showed to be a significant predictor of state emotional exhaustion [*γ* = 0.42, *SE* = 0.06, *t*(106) = 7.53, *p* < 0.001]. Hypothesis 1a was supported as entering job control to the model (Model 2) significantly improved the model fit (Δ −2*log = 105.79, *p* < 0.001) and job control was significantly and negatively related to emotional exhaustion [*γ* = −0.13, *SE* = 0.03, *t*(110) = −4.76, *p* < 0.001]. Hypothesis 1c stated that more time pressure in the morning is related to more emotional exhaustion in the afternoon. Results supported this hypothesis as adding time pressure to Model 1 significantly improved the model fit (Δ −2*log = 96.63, *p* < 0.001), and time pressure showed to be significantly and positively related to emotional exhaustion [*γ* = 0.12, *SE* = 0.02, *t*(110) = 4.81, *p* < 0.001]. To test hypotheses 3a and 3c, we first extended Model 2 by LMX (Model 3) and then the LMX by job control, respectively, the LMX by time pressure interaction (Model 4). There was no support for hypotheses 3a and 3c by the data as neither of the two models showed a better fit compared to Model 3 (for job control: Δ −2*log = 3.56, *p* > 0.50; for time pressure: Δ−2*log = 3.14, *p* > 0.50). Furthermore, neither the LMX by job control interaction [*γ* = −0.01, *SE* = 0.03, *t*(103) = −0.29, *p* = 0.78] nor the LMX by time pressure interaction [*γ* = 0.04, *SE* = 0.02, *t*(103) = 1.74, *p* = 0.08] significantly predicted emotional exhaustion.

#### Next-day job satisfaction as dependent variable

Hypotheses 2b and 2d stated that job characteristics on one day are related to morning job satisfaction on the next day. Compared to the null model predicting next day morning job satisfaction, the model fit improved by adding control variables (Δ −2*log = 86.67, *p* < 0.001). However, only the trait job satisfaction was significantly related to next day morning job satisfaction [*γ* = 0.58, *SE* = 0.06, *t*(107) = 9.05, *p* < 0.001]. Adding job control as a predictor to that model resulted in an improved model fit (Δ −2*log = 457.42, *p* < 0.001), but counter to hypothesis 2b job control on one day did not significantly predict job satisfaction on the following day [*γ* = 0.02, *SE* = 0.05, *t*(109) = 0.44, *p* = 0.66]. Adding time pressure to the control variables-only model as well led to a better model fit (Δ −2*log = 429.46, *p* < 0.001) but the predictor also did not reach significance [*γ* = −0.02, *SE* = 0.04, *t*(109) = −0.41, *p* = 0.68]. Therefore, hypothesis 2d could not be supported. To test hypotheses 4b and 4d, LMX was added to the model first. Second, the model was extended by the LMX by job control interaction, respectively, the LMX by time pressure interaction to predict next day job satisfaction. Adding the interaction term to the previous model did not improve the model fit for either of the models (for job control: Δ −2*log = 2.63, *p* > 0.50; for time pressure: Δ−2*log = 2.62, *p* > 0.50). The interaction terms did not reach significance [for the LMX by job control interaction: *γ* = −0.05, *SE* = 0.05, *t*(98) = −0.98, *p* = 0.33; for the LMX by time pressure interaction: *γ* = −0.02, *SE* = 0.04, *t*(98) = −0.44, *p* = 0.66]. Therefore, there was no support for hypotheses 4b and 4d.

#### Next-day emotional exhaustion as dependent variable

Hypotheses 2a and 2c stated that job characteristics on one day are related to morning emotional exhaustion on the next day. Compared to the null model predicting next day morning emotional exhaustion, the model fit improved by adding control variables (Δ −2*log = 52.33, *p* < 0.001). However, only trait emotional exhaustion was significantly related to next day morning job satisfaction [*γ* = 0.40, *SE* = 0.05, *t*(107) = 7.82, *p* < 0.001]. In model 2, job control on one day did not significantly predict emotional exhaustion on the following day [*γ* = 0.01, *SE* = 0.03, *t*(109) = 0.38, *p* = 0.71] even though adding the predictor to the previous model resulted in a better model fit (Δ −2*log = 305.66, *p* < 0.001). The same turned out to be true for time pressure as a predictor of next-day emotional exhaustion: The model fit improved (Δ −2*log = 294.24, *p* < 0.001), but the predictor did not reach significance [*γ* = 0.04, *SE* = 0.03, *t*(109) = 1.29, *p* = 0.20]. Thus, neither hypothesis 2a nor 2c was supported by the data. To test hypotheses 4a and 4c, the same model test, as described above, was used with the only difference that next day emotional exhaustion was the dependent variable. Neither did adding the interaction terms to the respective model result in a better model fit (for job control: Δ −2*log = 2.41, *p* > 0.50; for time pressure: Δ −2*log = 3.08, *p* > 0.50) nor did the interaction terms reach statistical significance in predicting next day emotional exhaustion [for the LMX by job control interaction: *γ* = −0.01, *SE* = 0.04, *t*(98) = −0.4, *p* = 0.69; for the LMX by time pressure interaction: *γ* = 0.00, *SE* = 0.03, *t*(98) = 0.12, *p* = 0.91]. Thus, data did not support hypotheses 4a and 4c.

### Additional analyses

Based on the suggestion by a reviewer, we additionally tested our hypotheses on the next-day effects with afternoon job characteristics and afternoon LMX as predictors for next-day morning job satisfaction and emotional exhaustion. Analyzing Model 2, afternoon job control significantly predicted next-day morning job satisfaction [*γ* = 0.11, *SE* = 0.05, *t*(110) = 2.14, *p* < 0.05] and the model fit improved compared to the one with control variables only (Δ −2*log = 491.53, *p* < 0.001). However, when controlling for next-day morning job control, the significant association of afternoon job control and next-day morning job satisfaction disappeared. Analyzing Model 4, the fit did not improve compared to Model 3 (Δ −2*log = 4.89, *p* > 0.50) but the job control by LMX interaction significantly predicted next-day morning job satisfaction [*γ* = −0.11, *SE* = 0.04, *t*(102) = −2.51, *p* < 0.05], also when controlling for next-day morning job control. However, in Model 4 afternoon job control was no longer a significant predictor of next-day morning job satisfaction. Additionally, when controlling for next-day morning LMX the interaction was no longer significant. All other analyses did not produce different results compared to the ones with morning job characteristics and morning LMX.

## Discussion

### Summary of findings

Building on COR theory, we assumed that daily LMX would moderate the relationship between job control and time pressure with job satisfaction and emotional exhaustion, respectively. Additionally, we proposed lagged effects from job characteristics and LMX on one working day to job satisfaction and emotional exhaustion on the next day’s morning. The data partly confirmed our hypotheses. As expected, on days when the participants experienced greater job control in the morning, they also reported more job satisfaction and a lower level of emotional exhaustion in the afternoon. Additionally, on days when the participants perceived more time pressure in the morning, they reported a higher level of emotional exhaustion in the afternoon. Unexpectedly, perceived job satisfaction in the afternoon was lower rather than higher on days with more perceived time pressure in the morning. Daily LMX moderated the relationship between morning job control and job satisfaction in the afternoon. On days when the participants reported lower levels of job control but high-quality relationships with their leaders, they reported higher job satisfaction than on days when they had lower levels of job control and lower-quality relationships. We found no evidence of moderation effects for the other relationships and no support for lagged effects when using morning variables as predictors. However, supplemental analyses showed lagged effects of the interaction between afternoon job control and afternoon LMX on next-day morning job satisfaction.

### Theoretical implications

#### Daily job control and daily work-related outcomes

We found that the participants reported greater job satisfaction on days when they experienced greater job control, which confirmed our hypothesis. Our findings mirror cross-sectional studies showing that people with a higher degree of autonomy in their jobs report higher job satisfaction than those with a lower degree of autonomy (Loher et al., 1985). We go beyond existing research by showing that this relationship can also be found on a daily level, that is, on a within-person level. Recent studies have shown that job satisfaction varies within persons and across days (Podsakoff et al., 2019; McCormick et al., 2020); this cannot be explained by differences between persons or more stable job and organizational factors. In turn, such variations are attributable to experiences and events that fluctuate within persons. We discovered that daily fluctuations in employees’ job control levels are related to within-person fluctuations in job satisfaction. Therefore, the daily level of job control is one work-related experience that can explain those fluctuations in job satisfaction. Additionally, we acknowledge this finding as pointing to the boosting effect of job control on positive indicators of well-being (Sonnentag, 2015), which again underlines the fact that job control is an important resource for employees in their daily routines.

We also found that job control is closely related to emotional exhaustion as a negative well-being indicator. People feel less emotionally exhausted on days they perceive greater job control. Therefore, we identified job control as the daily-level variable that can help to explain fluctuations in employees’ emotional exhaustion. Hence, it seems as if a high daily level of job control not only enhances positive experiences but is also a resource associated with reduced daily feelings of being emotionally exhausted. In this way, we clarify a dynamic link between job control and negative well-being indicators that have rarely been studied (c.f., Sonnentag, 2015). In sum, we have complemented between-person findings regarding job control (Crawford et al., 2010; Nahrgang et al., 2011) with within-person findings because we have shown an increase in job control on one day relative to another is beneficial for employees.

#### Daily time pressure and daily work-related outcomes

In line with the challenge-hindrance stressor framework (LePine et al., 2005; Podsakoff et al., 2007), we assumed time pressure to be a challenge stressor that is beneficial for daily job satisfaction. However, daily time pressure was associated with lower job satisfaction. Especially in the light of our day-level perspective, this finding was surprising. We expected to find short-term positive effects of time pressure on job satisfaction because high time pressure might be associated with feelings of being challenged, with a greater number of completed tasks, or with increased self-esteem through goal achievement during the day (Widmer et al., 2012). We conclude that, even though days with elevated levels of time pressure can motivate and energize employees (Sonnentag, 2015), and thus can be beneficial for work engagement (Sonnentag et al., 2012; Garrick et al., 2014), it seems detrimental to job satisfaction. One explanation for this can be found in appraisal theories (Lazarus and Folkman, 1984). These argue that it is not the objective stressor itself that has negative consequences but the appraisal of the stressor as being stressful; the feeling of not being able to cope with it then leads to negative consequences. In particular, a stressor is beneficial only if it is appraised as challenging. The individual must be sure that they will be able to cope with it, for example, by putting more effort into a task. Research showed that challenge appraisals mediated the relationship of daily time pressure on daily creativity and proactive behaviors (Ohly and Fritz, 2010). Their results were confirmed by another diary study that found that distinct appraisals of stressors were related differently to well-being (Tuckey et al., 2015). As challenge appraisals were not assessed in the present study, we encourage future researchers to address the issue by explicitly measuring challenge appraisals. One might assume that time pressure is positively related to job satisfaction, but only when time pressure is appraised as challenging.

A second aspect concerns our short-term, day-level perspective. Even though we assessed time pressure on the day level and therefore assessed the momentary state level of time pressure, we acknowledge that the daily evaluation of the degree of time pressure can be shaped by factors that are not bound to that day.^2^ In particular, past experiences in the days or weeks before our assessment may have influenced the participants’ perceptions and answers. For example, a person who experienced much time pressure in the weeks before our study is likely to rate the day-specific level of time pressure differently than someone who faced relatively little. This situation makes it difficult to differentiate between short-term and long-term effects, as Baethge et al. (2018) suggested. It is, therefore, necessary to consider the duration of experienced time pressure (Kunzelmann and Rigotti, 2021). Again, we encourage future research to consider the general level of time pressure as a contextual variable that can influence how people react to elevated levels. Scholars recently showed that daily and general levels of job characteristics interact in predicting work-related outcomes (Oerlemans and Bakker, 2018).

Third, connected to the two aspects mentioned above, a recent study found that challenge stressors are only positively related to employee performance and well-being when they experience a stable pattern of challenge stressors over time (Rosen et al., 2020). As argued by the authors, they are more predictable when challenge stressors are stable. In turn, employees are more likely to be able to deal with them effectively. In contrast, unstable challenge stressors are less predictable and associated with increased anxiety. Therefore, future studies might benefit from incorporating the degree of stability of challenge stressors to receive a more fine-grained picture of circumstances for positive or negative associations with job satisfaction.

We also found support for a positive association between daily time pressure and feelings of emotional exhaustion. We argued that employees might put extra cognitive or mental effort into their task to deal with the former, which in turn is connected to psychological costs later that day, such as exhaustion (Demerouti et al., 2001). Therefore, the results of our study confirm LePine et al.’s (2005) and Pindek et al.’s (2019) findings, that is, that challenge stressors (e.g., time pressure) lead to strain and exhaustion, as well as diary studies showing that dealing with higher levels of job stressors than usual is associated with increased negative indicators of well-being (e.g., Zohar et al., 2003; Rodell and Judge, 2009). Therefore, we have shed light on the double-edged sword of time pressure for employees.

#### Moderating role of daily LMX

To the best of our knowledge, the present study is the first to investigate the moderating role of daily LMX on the link between job characteristics and work-related outcomes. Our data confirmed the hypothesis that daily perceptions of a high-quality LMX buffer the negative link between low job control and job satisfaction. This finding mirrors the literature that has shown how important high-quality LMX is in helping employees to cope with stressors (Hackney et al., 2018; Zhang et al., 2019). In line with COR theory (Hobfoll, 1989), LMX seems to be a work-related social resource that helps to protect valuable resources (e.g., health or well-being) when facing actual or impending resource loss. As resource loss has a much greater impact than resource gain (Hobfoll et al., 2018), our study shows that leaders play an important role in maintaining employee job satisfaction by buffering the negative link with (perhaps unchangeable) working conditions.

Our findings build on previous research that indicated that LMX is a useful resource for employees. In contrast to prior studies investigating LMX as a moderator (e.g., Hackney et al., 2018; Zhang et al., 2019), we examined its immediate, daily effects. We discovered that employees’ daily perceptions of the quality of LMX play a role in buffering the reduced job satisfaction associated with a low level of job control (Volmer, 2015; Ellis et al., 2019). Hence, the perception of how supportive the leader is *today* concerning today’s tasks, for example, by giving advice and guidance on a work task (Wayne et al., 1997) or assisting in sensemaking processes (Harris and Kacmar, 2006), is important in maintaining job satisfaction when employees are facing tasks with low job control.

Even though our hypothesis was supported by the data concerning the job control–job satisfaction link, the moderating role of LMX was not observed when emotional exhaustion was used as an outcome. We assume that even though leaders might mitigate how employees cope with unfavorable task characteristics (e.g., low job control), the unfavorable task characteristic and the associated strain might be less tangible to the leader. We assume that the task’s nature depletes physical, psychological, or cognitive resources and is therefore linked to emotional exhaustion. Following the control model of demand management (Hockey, 1995), environmental stressors make people apply performance-protection strategies by actively controlling information processing, which increases subjective effort (Demerouti et al., 2001). Consequently, task performance is more demanding, reflected in a greater level of emotional exhaustion.

Additionally, we could not detect the moderating role of LMX when using time pressure as a predictor. One possible explanation might be found in the consequences of a very high-quality LMX. Some authors have suggested that it could be too much of a good thing (Harris and Kacmar, 2006). They demonstrated a curvilinear relationship between LMX and stress; in other words, stress was highest in either very low-or very high-quality LMX relationships. The authors argued that employees in high-quality relationships might feel obliged to put extra effort into their work to meet the leader’s expectations and not disappoint them. Following this argument, time pressure would not decrease but increase when LMX quality increases. In line with the multidimensional concept of LMX (Liden and Maslyn, 1998) the individual contribution and commitment to a shared goal might make an employee go the extra mile for the leader. Therefore, one may gather that high-quality LMX is not a job resource but a job stressor in certain situations. Future studies might clarify which situational characteristics lead to perceptions of LMX as either a stressor or a resource.

Our results should be interpreted against the background of recent criticism of the LMX construct (Gottfredson et al., 2020; Scandura and Meuser, 2022). First, LMX was criticized for lacking a clear definition. For example, it is unclear whether LMX captures a role-defining process of followers, the quality of dyadic exchanges between a leader and a follower, or the follower’s perception of the relationship quality with their leader. Furthermore, it is unclear what exactly is meant by *quality of exchange relationship*, that is, whether there is a distinction between single exchange activities and an overall rating of the relationship quality (Scandura and Meuser, 2022). Additionally, the LMX construct has been attached to different theories, which is why the theoretical basis of LMX and what the construct captures are unclear. Second, the common-used LMX-7 scale was criticized for lacking methodological clarity in the development and validation, and, connected to the discussion above, it is used to measure the multiple definitions of the LMX construct. Due to the unclear conceptualization of the construct and the measurement, it was also criticized that the level of analysis (i.e., follower-, leader-, or dyad-level) is unclear and misaligned with the conceptualization and measurement. Thus, we acknowledge that we cannot be specific on what exactly happened when LMX was higher or lower than usual. We could not consider specific resources exchanged between leader and follower (Wilson et al., 2010). Additionally, LMX is an endogenous construct that is influenced by other variables (Gottfredson et al., 2020). For example, stable and day-specific attributes and behaviors of both the leader and the follower influence how the follower rates the LMX items. Knowledge of these attributes and behaviors would have been helpful to be more specific on factors that can help to explain the buffering role of LMX in situations with lower job control. Taken together, our finding of the moderating role of LMX in the job control-job satisfaction association should be interpreted with caution in light of a follower-based evaluation of the leader-follower relationship, which might resemble an overall rating of interactions on a specific day.

#### Lagged effects of daily job characteristics on next-day work-related outcomes

We were surprised not to find any direct lagged effects. Previous studies (e.g., Fritz and Sonnentag, 2009; Nicholson and Griffin, 2015) showed that events on one day are associated with next-day variables. However, we could not control activities and events between the afternoon assessment on one day and the morning assessment on the next. The literature has suggested that sufficient recovery in the evening can compensate for stressful work events during the day, which gives employees the chance to start the next day with fully recharged resources (Bennett et al., 2018; c.f., Sonnentag et al., 2017). As we would not rule this out, we would advise future researchers to include quantitative or qualitative measurements of recovery experiences when studying the lagged effects of job characteristics on work-related outcomes. Previous research has demonstrated that temporary mood accounts for almost one-third of the within-person variance in job satisfaction, and job satisfaction and mood vary concurrently (Ilies and Judge, 2002). There might have occurred other events that were independent of the job characteristics experienced on one day. For example, events in the evening (e.g., an experienced work–family conflict or pleasurable evening activities) or on the next morning (e.g., positive or negative feedback on one’s work) may have been more important for next-day employee job satisfaction and emotional exhaustion than the previous day’s job characteristics. However, the finding of our additional analyses that the interaction of afternoon job control and LMX was related to next-day morning job satisfaction might suggest that the buffering effect of LMX can also transfer to the next day.

### Practical implications

First, we identified high levels of job control and low levels of time pressure on a specific day as two relevant job-related experiences that can explain increases in job satisfaction and decreases in emotional exhaustion on a daily level. Thus, to facilitate higher levels of employee job satisfaction and lower levels of emotional exhaustion, one option available to organizations and leaders is to give employees control and autonomy over how they perform their tasks. Leaders can provide this by upskilling and trusting their employees, sharing necessary information, and stressing the importance of results rather than the way tasks are performed. Our study additionally suggests that higher daily levels of job control are even beneficial for employees who (on average) experience high levels of job control (as was the case in our sample). That means that organizations, leaders, and employees should strive to find ways to increase job control for every task and working day whenever possible. However, leaders should remember that the ideal degree of autonomy might vary between individuals; high levels of job control could be overwhelming for some employees, especially when confronted with new tasks.

Our findings indicate that practitioners should keep in mind the positive link between time pressure and emotional exhaustion and aim to lower time pressure on a daily basis. Time pressure did not emerge as a typical challenge stressor and had no beneficial effects on job satisfaction. Therefore, increasing time pressure even on single days is insufficient to facilitate job satisfaction. A reduction of time pressure at work can be enhanced by leader behavior, for instance, by not demanding unnecessarily strict deadlines and by equipping employees with time management strategies. Employees can try to reduce time pressure, for example, by not multitasking, communicating with their leader regarding workloads, and rethinking their work style. However, even though the present study has pointed to a linear relationship between time pressure and job satisfaction and emotional exhaustion, a medium level of time pressure might in some individuals enhance well-being (Reis et al., 2017).

Second, our study showed substantial day-to-day variance in the constructs under discussion. Almost half (46%) of the total variance in LMX was within-person, suggesting that LMX fluctuates daily (c.f., Ellis et al., 2019). Consequently, leaders should not rely on the quality of the relationship they have built up because perceptions of it can change. We learned that a negative association of low job control with job satisfaction could be constrained by an employee’s perception of a high-quality relationship with the leader on a specific day. Therefore, leaders should not underestimate their role in their employees’ job satisfaction and should be aware of the buffer effect of high-quality LMX. Additionally, our investigation into daily LMX should encourage leaders to regularly reflect on the quality of their relationships with each follower and provide them with sufficient resources whenever necessary. The buffer effect might be especially significant in situations where job characteristics such as job control cannot (easily) be changed, for example, when there are no or only a few degrees of freedom to perform a task. By demonstrating attention and loyalty, leaders can give their employees the feeling that they are being acknowledged. They can also try to make sense of situations perceived as threats (Harris and Kacmar, 2006) and provide task guidance (Wayne et al., 1997).

### Limitations

Even though we consider our study to have several strengths (e.g., the limited risk of retrospective bias, the temporal separation of predictor and outcome, and the investigation of within-person effects), it also has some limitations.

First, we used employee self-ratings. While these are appropriate for measuring perceptions and internal states, which we were most interested in, we could not rule out the possibility of common-method bias (Podsakoff et al., 2012). The dyadic nature of LMX implies that leaders’ views of the quality of their relationship with employees should also be taken into account; any overlaps could be examined and hopefully acted upon (though research has shown that perceptions of the quality of LMX might differ between leaders and employees; Sin et al., 2009). Future studies should therefore include leaders’ perceptions of LMX and build on research (Schyns and Day, 2010) to demonstrate that “the amount of agreement itself embraces important information about the relationship quality” (Sonnentag and Pundt, 2016, p. 190).

Second, the study design did not allow for causal inferences because we did not experimentally manipulate or control for other variables. However, we introduced a temporal dimension by assessing the predictor variables (morning) and the dependent variables (afternoon) at different times of the day. Temporal separation of the measurement of the predictor and the criterion variables has been suggested as a way of controlling for artificially inflated relationships (Podsakoff et al., 2003).

Third, the sample consisted of scientific employees. Such individuals, especially those working in universities, usually enjoy a very high degree of autonomy (as was evidenced in the present study, where the mean job control score was four on a one-to-five scale) and may have only infrequent contact with their leaders. Therefore, the generalizability of the findings might be limited. Future researchers could investigate the moderating role of LMX in a more varied sample (e.g., in terms of job control, time pressure, and frequency and significance of contact with leaders).

Last, we only investigated two job characteristics (i.e., job control and time pressure). However, there might be other daily job demands (e.g., emotional demands, conflicts at work) and resources (e.g., support from coworkers, feedback) that we did not assess, and that can help to explain fluctuations in daily job satisfaction and emotional exhaustion. Connected to this, we did not control for other daily job demands and resources, which potentially results in an overestimation of our effects. We encourage future research to investigate these alternative daily predictors of job satisfaction and emotional exhaustion.

## Conclusion

The present study makes two important contributions to the literature on job characteristics, leadership, and work-related outcomes. First, it complements current day-level resource-based research by demonstrating that daily perceptions of high job control are relevant for both motivational and affective outcomes and health-relevant outcomes (i.e., emotional exhaustion). Additionally, it shows how daily perceptions of high time pressure relate not only to health-associated but also to affective and attitudinal outcomes (i.e., job satisfaction). Second, we extend the literature on LMX that regards LMX as a work-related social resource for employees by demonstrating that daily LMX acts as a buffer on the job control–job satisfaction nexus. Therefore, our study suggests that, if possible, jobs should be designed for employees’ benefit and that leaders can buffer negative consequences when their staff is dealing with unfavorable working conditions.

## Data availability statement

The raw data supporting the conclusions of this article will be made available by the authors, without undue reservation.

## Ethics statement

Ethical review and approval was not required for the study on human participants in accordance with the local legislation and institutional requirements. The patients/participants provided their written informed consent to participate in this study.

## Author contributions

JV designed the study and collected the data. LP and JV developed the research question and hypotheses. LP conducted the analyses and discussed the results with JV. LP wrote the manuscript in consultation with JV. All authors contributed to the article and approved the submitted version.

## Funding

This research was funded by the Commission for Research and Young Scientists (Ständige Kommission für Forschung und wissenschaftlichen Nachwuchs (FNK)) of the University of Bamberg. The authors acknowledge support from the Open Access Publication Fund of the University of Bamberg.

## Conflict of interest

We declare that the research was conducted in the absence of any commercial or financial relationships that could be construed as a potential conflict of interest.

## Publisher’s note

All claims expressed in this article are solely those of the authors and do not necessarily represent those of their affiliated organizations, or those of the publisher, the editors and the reviewers. Any product that may be evaluated in this article, or claim that may be made by its manufacturer, is not guaranteed or endorsed by the publisher.

## Footnotes

^1^We thank an anonymous reviewer for this note.

^2^We thank an anonymous reviewer for raising this issue.
